# Dissecting Selective Signatures and Candidate Genes in Grandparent Lines Subject to High Selection Pressure for Broiler Production and in a Local Russian Chicken Breed of Ushanka

**DOI:** 10.3390/genes15040524

**Published:** 2024-04-22

**Authors:** Michael N. Romanov, Alexey V. Shakhin, Alexandra S. Abdelmanova, Natalia A. Volkova, Dmitry N. Efimov, Vladimir I. Fisinin, Liudmila G. Korshunova, Dmitry V. Anshakov, Arsen V. Dotsev, Darren K. Griffin, Natalia A. Zinovieva

**Affiliations:** 1 L. K. Ernst Federal Research Center for Animal Husbandry, Dubrovitsy, Podolsk 142132, Moscow Oblast, Russia; alexshahin@mail.ru (A.V.S.); abdelmanova@vij.ru (A.S.A.); natavolkova@inbox.ru (N.A.V.); asnd@mail.ru (A.V.D.); 2 School of Biosciences, University of Kent, Canterbury CT2 7NJ, UK; d.k.griffin@kent.ac.uk; 3 Federal State Budget Scientific Institution Federal Scientific Center “All-Russian Research and Technological Poultry Institute”, Sergiev Posad 141311, Moscow Oblast, Russia; dmi40172575@gmail.com (D.N.E.); olga@vnitip.ru (V.I.F.); lg@vnitip.ru (L.G.K.); 4 Breeding and Genetic Center “Zagorsk Experimental Breeding Farm”—Branch of the Federal Research Center “All-Russian Poultry Research and Technological Institute”, Russian Academy of Sciences, Sergiev Posad 141311, Moscow Oblast, Russia; a89265594669@rambler.ru

**Keywords:** selection signatures, genomic regions, candidate genes, chicken, SNPs, White Cornish breed, Plymouth Rock White breed, Ushanka breed, genetic diversity, broiler production

## Abstract

Breeding improvements and quantitative trait genetics are essential to the advancement of broiler production. The impact of artificial selection on genomic architecture and the genetic markers sought remains a key area of research. Here, we used whole-genome resequencing data to analyze the genomic architecture, diversity, and selective sweeps in Cornish White (CRW) and Plymouth Rock White (PRW) transboundary breeds selected for meat production and, comparatively, in an aboriginal Russian breed of Ushanka (USH). Reads were aligned to the reference genome bGalGal1.mat.broiler.GRCg7b and filtered to remove PCR duplicates and low-quality reads using BWA-MEM2 and bcftools software; 12,563,892 SNPs were produced for subsequent analyses. Compared to CRW and PRW, USH had a lower diversity and a higher genetic distinctiveness. Selective sweep regions and corresponding candidate genes were examined based on Z*F*_ST_, hapFLK, and ROH assessment procedures. Twenty-seven prioritized chicken genes and the functional projection from human homologs suggest their importance for selection signals in the studied breeds. These genes have a functional relationship with such trait categories as body weight, muscles, fat metabolism and deposition, reproduction, etc., mainly aligned with the QTLs in the sweep regions. This information is pivotal for further executing genomic selection to enhance phenotypic traits.

## 1. Introduction

Broiler production is both one of the leading and fastest-growing parts of the worldwide food production industry [1,2,3,4]. By 2031, 153.85 metric kilotons of poultry meat are anticipated to be consumed globally and 41% of all world meat consumption will be chicken (as reviewed by [2]). The progress in broiler production relies upon advances in selective breeding [5] and the genetics of quantitative traits [6,7]. The respective influence of the latter on reproductive fitness has been, and continues to be, a major subject of study in artificial selection experiments [8,9]. It is well recognized that long-term artificial selection of animals affects the genomic architecture of breeds and yields genetic signatures for breeding traits [10]. More about the dynamics of advantageous (and disadvantageous) alleles arising from the founder population, or appearing as novel mutations under continuing selection, can be learnt by tracking genomic changes through time in populations that have been subjected to intense artificial directional selection [11,12,13,14,15]. Such studies are a key source of knowledge in exploring how selection affects the genome and quantitative trait loci (QTLs). In this vein, domesticated chicken breeds can be considered as long-term artificial selection experiments. One such example is the famous bidirectional selection experiment for body weight in the Virginia chicken lines that started in 1957 [16,17,18,19,20]. Continual monitoring of the existing poultry genetic stocks from these breeds and lines is crucial for their sustainability and use in commercial breeding programs (e.g., [21,22,23,24,25,26,27]).

**Figure 1:**
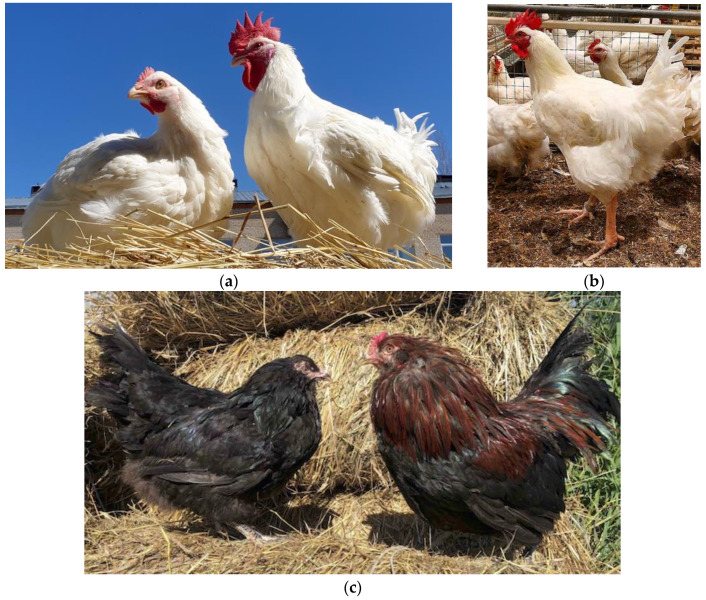
The three chicken breeds examined in this study. (**a**) Cornish White (female, left; male, right); (**b**) Plymouth Rock White (male, front; females, back); and (**c**) Ushanka (female, left; male, right).

A representative proportion of segregating variation in breeding experimental data available from designs of commercial broiler crosses using parent strains of divergent artificial selection experiments is higher than that produced by crossing two random inbred lines [28,29,30]. This principle is used in these cross designs and consists of four grandparent lines: two (paternal and maternal) for producing a male parent (breeder) stock and two (paternal and maternal) for producing a female parent (breeder) stock (e.g., [31,32,33]). Usually, two transboundary meat-type breeds, Cornish White (CRW; Figure 1a) and Plymouth Rock White (PRW; Figure 1b), are used in commercial broiler production as male and female breeder stocks, respectively [34,35,36,37].

By selecting for characteristics such as rapid growth/development, feed efficiency, and high yield/quality of breast meat, it is possible to enhance the genetic potential of broiler chickens further [31,38]. This can be achieved through studies of association between candidate genes and phenotypic traits in commercial broiler (breeder) populations [39,40,41,42,43,44,45,46]. Quantitative genetics, computer science, and DNA chip technologies are used in broiler breeding operations to select breeding birds. In pedigree selection systems, significant genetic markers such as single nucleotide polymorphisms (SNPs) can be identified through the use of DNA chip technology, as well as more novel marker-assisted and genomic selection strategies [31,47,48,49]. Previously, genome-wide SNP scans have been used efficiently for studying the genetic architecture, diversity, selective footprints, and evolutionary implications in various Russian meat-type and other chicken breeds and lines [50,51,52,53,54,55,56,57,58,59,60,61,62,63]. Whole-genome resequencing approaches can produce even more SNPs that are usable for in-depth analyses of genomic architecture and candidate genes [20,64,65].

The purpose of the present study was to perform whole-genome resequencing and examine in more detail the selection trajectories in the genomes of the pure grandparent lines of two transboundary meat-type breeds. These were CRW (Figure 1a) and PRW (Figure 1b) that are used in a four-way broiler cross developed in Russia. As a comparison group, we chose the primitive dual-purpose and fancy breed named Ushanka (USH; Figure 1c) that has been bred in Russia for a few centuries in a closed population without any strict selection pressure [59,62,63].

## 2. Materials and Methods

### 2.1. Experimental Animals, Sample Collection, and DNA Extraction

The sampling of animals examined included 20 CRW, 20 PRW, and 17 USH male birds. The transboundary CRW and PRW breeds were represented by birds of the four-way broiler cross Smena 8 [66,67] developed at the Breeding and Genetic Center “Smena” (BGCS; Bereznyaki, Moscow Oblast, Russia), Branch of the Federal Scientific Center “All-Russian Research and Technological Poultry Institute” (FSC ARRTPI; Sergiev Posad, Moscow Oblast, Russia). Within the CRW breed there were two lines: B5 (the paternal line of the male parent stock of this cross) and B6 (the maternal line of the male parent stock). Similarly, for PRW, there were two lines: B7 (female parent stock’s paternal line) and B9 (female parent stock’s maternal line). For each of the above four grandparent lines, 10 male birds were sampled. USH is known as an archaic indigenous breed that manifests cold adaptation and is bred in a confined population with little or no selection pressure [62,63]; it was used as a comparative group in the present investigation.

The CRW and PRW chickens were provided by BGCS and those of the USH breed by the Breeding and Genetic Center “Zagorsk Experimental Breeding Farm” (Sergiev Posad, Moscow Oblast, Russia), FSC ARRTPI Branch. The breed flocks were housed in the bioresource Gene Pool Collection of Farm and Wild Animals and Birds at the L. K. Ernst Federal Research Center for Animal Husbandry (LKEFRCAH, Dubrovitsy, Moscow Oblast, Russia). All of the experimental birds had a basic feed and maintenance conditions that complied with zootechnic and zoohygienic norms stated elsewhere (e.g., [68,69]).

Samples of pulp-containing feathers were taken from 57 chickens of all three breeds and lines studied. The Syntol kit for DNA isolation from animal tissues (Syntol LLC, Moscow, Russia) was used to extract DNA. The concentration of the DNA solution was measured with a Qubit 3.0 fluorimeter (Thermo Fisher Scientific, Wilmington, DE, USA). Using a NanoDrop-2000 device (Thermo Fisher Scientific), the OD260/280 ratio was measured to verify the extracted DNA’s purity.

### 2.2. Sequencing, SNP Genotyping and Quality Control

The samples collected were sequenced using paired-end sequencing (2 × 150 bp) and an Illumina NextSeq instrument (San Diego, CA, USA), with a mean coverage of 20×.

Filtering of whole-genome resequencing raw data was carried out using the Fastp program [70], with the recommended launch parameters. During the filtering process, nucleotide sequences of Illumina adapters and sequences with low-quality reads were trimmed. Quality control before and after using the Fastp program was carried out using the FastQC program [71]. Mapping of short reads to the reference genome was carried out using the BWA-MEM2 software package [72] based on the bwa-mem algorithm of the original BWA program [73]. The chicken (*Gallus gallus*; GGA) assembly bGalGal1.mat.broiler.GRCg7b (Ensembl release 108) [74,75] was used as a reference genome. Sorting, removing duplicates, and indexing the resultant files in BAM format were carried out using the samtools set of utilities [76]. Determination of SNP positions, insertions and deletions, and manipulation of VCF files were performed using the bcftools package [77]. The Tabix program [78] was also used to index VCF files. The GNU Parallel program [79] was used to execute tasks in parallel in order to reduce calculation time.

The generated number of reads per breed was 308.24 ± 10.85 million, totaling 44.40 ± 0.88 GB. An average sequence coverage was 21.07 ± 0.42 X. A total of 12,563,892 polymorphic SNPs were selected for further analysis. Hereby, sex chromosome (GGAZ and GGAW) SNPs were excluded from the analysis.

### 2.3. Genetic Diversity and Population Structure

Analysis of genetic diversity and interbreed relationships was performed as described in [58]. In brief, to assess genetic diversity within populations, PLINK v1.9 software was employed [80,81]. Principal component analysis (PCA) based on the variance-standardized relationship matrix was performed using PLINK [80], and the results were visualized using the R package ggplot2 [82]. R package diveRsity [83] was used to calculation observed heterozygosity (*H_O_*), unbiased expected heterozygosity (*_U_H_E_*) [84], rarefied allelic richness (*A_R_*) [85], and inbreeding coefficient (*_U_F*_IS_) based on the unbiased expected heterozygosity.

The genetic admixture analysis of the populations studied was performed using Admixture v1.3 software [86,87], and the results were plotted using the R package BITE [88]. The number of ancestral populations (K) was determined using a conventional admixture cross-validation (CV) approach [89]. When compared to different K numbers, the assumed number of K conformed to the CV error value that was lowest (Supplementary Figure S1).

### 2.4. Genetic Diversity and Population Structure

#### 2.4.1. Z*F*_ST_ Estimation

We analyzed population differentiation based on mean *F*_ST_ values over a 50 kB sliding window with 10 kB steps, assuming that changes during selection pressure affect not only the target region but also its associated sites. The window size was chosen based on the degree of attenuation of linkage disequilibrium (LD) toward the genome-wide background in order to set the window to roughly the size where LD decays to the genome-wide background (Supplementary Figure S2).

LD decay was calculated with PLINK using the original script (as described in [90]). To limit false-positive outliers, the mean *F*_ST_ values were Z-transformed to generate Z*F*_ST_ values as follows: Z*F*_ST_  =  (*F*_ST_ − *μF*_ST_)/*σF*_ST_, where *μF*_ST_ and *σF*_ST_ are the mean and standard deviation of *F_ST_* values in all windows [64,91]. In fact, Z*F*_ST_ values indicate the number of standard deviations of the *n*th value from the mean. That is, they are suitable to search for outliers in a data array because they relate to the conventional values of ±3*σ* that include 99.7% of values with a normal distribution. In our case, we specifically looked for variants of *F*_ST_ values that deviate greatly from the mean. Regions containing SNPs for which Z*F*_ST_ values were included in the 0.1% of maximum values were considered to be the areas most subject to selection pressure.

#### 2.4.2. HapFLK Procedure

To detect the signatures of selection through haplotype differentiation among the studied breeds, we also employed the hapFLK 1.4 program [92,93]. In fastPHASE, the number of haplotype clusters per chromosome was established at 35 by the use of cross-validation-based estimation [94,95,96,97,98]. We chose the hapFLK areas with at least one SNP and a *p*-value cutoff of 0.00001 (−log10(*p*) > 5) for in-depth analysis.

#### 2.4.3. ROH and Inbreeding Estimation

We used a *consecutiveRUNS.run* function [99,100,101,102] implemented in the R package detectRUNS [103,104,105] for estimation of runs of homozygosity (ROH) [106,107]. To avoid the inclusion of the most common short fragments in the results, we set the minimum length for ROH to 0.5 MB. Considering that the density of genomic data is significantly higher than that of SNP-chip data, the values of a possible missing genotype and a possible heterozygous genotype (maxMissRun and maxOppRun) [108,109,110] were taken as 21. The latter value was obtained as the ratio of the density of our data (12.5 M SNPs) to the densest SNP chip for chickens (600 K SNPs). We determined the minimal number of SNPs (*l*) as was first assessed by Lencz et al. [111] and later modified by Purfield et al. [112] in order to minimize false-positive outcomes. In our study, the minimum number of SNPs was equal to 50. The respective genomic inbreeding coefficient (*F*_ROH_) was computed using data regarding the homozygous region count and length in the examined breed genomes [113]. This *F*_ROH_ estimate was represented by the proportion of each individual’s total length of ROH to the length of the autosomal SNP-covered reference genome [62,114,115].

### 2.5. Detection of Candidate Genes and QTLs in Selective Sweep Regions

The web-based Ensembl Genes release 103 database and Ensembl BioMart data mining tool [116,117,118,119] were utilized to retrieve chicken genes and their human orthologs based on the boundaries of these regions as located in the GRCg7b reference assembly chromosomes. To find primary candidate genes and other genes of interest, results for each genomic area of selection signature that were obtained from the Ensembl BioMart browser were manually sifted and compared to pertinent published studies. The genes from the regions supported by at least two different techniques were considered as prioritized candidate genes (PCGs).

QTLs that are localized in the genomic regions of interest and can contain candidate genes were searched using an in-house R script. Herewith, we identified the boundaries of the region of interest matching to QTL genome location using a downloaded copy of the Chicken QTLdb database [120,121].

## 3. Results

### 3.1. Between- and Within-Breed Genetic Diversity

**Figure 2:**
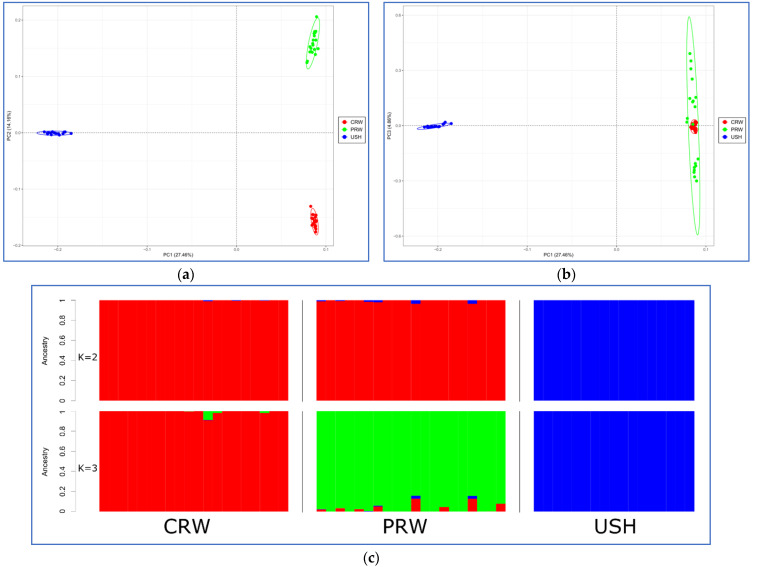
Genetic relationships among the three chicken breeds studied using genome-wide SNP genotyping. (**a**,**b**) PCA plots showing the distribution of breeds and individuals in the dimensions of two coordinates, i.e., the first (PC1; *X*-axis) and second (PC2; *Y*-axis; (**a**) or third (PC3; *Y*-axis; (**b**) principal components; (**c**) admixture-based bar plots illustrating the proportions of individual ancestry in the breeds under study at K = 2 (**top**) and K = 3 (**bottom**). Breeds: CRW, Cornish White; PRW, Plymouth Rock White; USH, Ushanka.

PCA revealed that the three studied breeds formed the appropriate breed-specific clusters (Figure 2a,b). Moreover, the USH chickens, being separated from the two meat-type breeds, demonstrated the lowest genetic variability based on their scattering on both PCA plots. The individuals that made up the PRW sample were more diverse than CRW and USH. The admixture analysis resulted in the optimal number of ancestral populations at K = 3 (Supplementary Figure S1), suggesting also that a few CRW and PRW individuals reflected admixtures from the other breeds in this dataset, while such admixtures were absent in USH (Figure 2c).

As follows from the data in Table 1, values of *A_R_*, *_U_H_E_*, and *H_O_* in the aboriginal USH breed were significantly lower (1.6218 ± 0.0004, 0.2068 ± 0.0001, and 0.2103 ± 0.0002, respectively; *p* < 0.001) than in both transboundary breeds CRW and PRW, for which these indicators differed slightly. The *_U_F*_IS_ inbreeding coefficients of CRW and PRW were, however, four times higher than that of USH. PRW was superior in *A_R_* to both USH and CRW, but in terms of *H_O_*, significant differences were found only for USH. A greater *A_R_*-based heterogeneity identified for PRW was confirmed by the above PCA results.

### 3.2. Signatures of Selection

#### 3.2.1. Z*F*_ST_ Statistic at Pairwise Comparison of Breeds

We searched for genomic regions that were established in the studied breeds under the influence of natural or artificial selection. These regions were identified by estimating the largest average *F*_ST_ for a sliding window. The analysis was carried out for each pair of breeds separately (Supplementary Figure S3, Supplementary Table S1). The analysis did not include the GGAZ and GGAW sex chromosomes.

As can be seen from Table 2, a pairwise comparison of CRW with two other breeds revealed six genomic regions containing three genes. For PRW and USH, there were nine and 11 identified regions containing two and four genes, respectively.

#### 3.2.2. HapFLK Statistic

**Figure 3:**
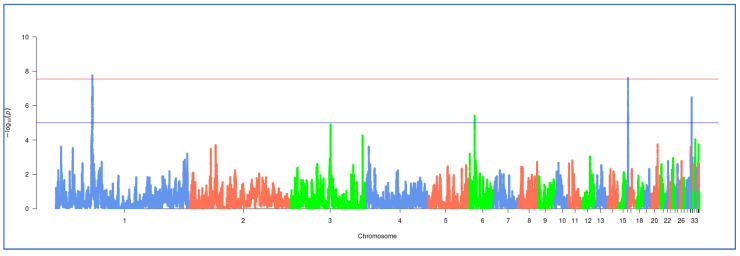
Search for signatures of selection in genomes of the studied breeds as revealed by the hapFLK analysis. Chicken autosomes are the values for the *X*-axis, and statistical significance values (−log_10_ *p*-values) are the values for the *Y*-axis. The red line that indicates the threshold of significance at *p* < 2.8 × 10^−8^ (i.e., −log_10_(*p*) > 7.55) was determined using the Bonferroni correction and defines the strongest hapFLK regions, while the blue line indicates the threshold of significance at *p* < 1 × 10^−5^ (i.e., −log_10_(*p*) > 5) and defines the putative hapFLK regions.

The hapFLK analysis was carried out for the combined sample of the three breeds. The results are visualized as a Manhattan plot with two threshold values (Figure 3).

The results of the hapFLK analysis for the three-breed dataset included four genomic regions on chromosomes GGA1, GGA6, GGA16, and GGA31, within or near which a total of 68 genes were localized (Table 3, Supplementary Figure S4, Supplementary Table S2).

#### 3.2.3. ROH Islands Detection

Within each breed, we established that over 50% of samples had overlapping ROH islands (Supplementary Table S3, Supplementary Figure S5, Table 4). A total of 261 homozygosity islands were discovered, which were localized on 19 chromosomes. The majority of ROH islands (95.40%) was of the USH breed.

**Figure 4:**
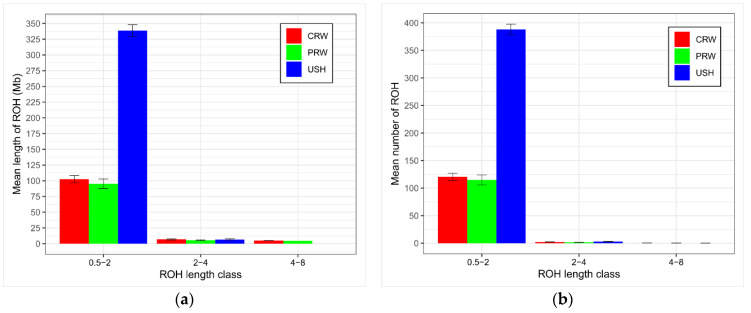
Descriptive statistics of the runs of homozygosity (ROHs) by ROH length class in the studied chicken breeds: (**a**) Overall mean length of ROHs (*Y*-axis) by ROH length class (*X*-axis; 0.5–2, 2–4 and 4–8 Mb). (**b**) Mean number of ROHs (*Y*-axis) by ROH length class (*X*-axis; 0.5–2, 2–4, and 4–8 Mb). Breeds: CRW, Cornish White; PRW, Plymouth Rock White; USH, Ushanka.

At the same time, the distribution analysis of the average length (Figure 4a) and the number of homozygosity segments by length class (Figure 4b) showed that USH, like two other studied breeds, are distinguished mainly by shorter segments, suggesting events of longstanding inbreeding. Herewith, the values of these indicators for USH significantly exceeded similar values for other breeds in the shortest length class; however, as the length of the ROH fragments increases, USH was inferior to CRW and PRW. Thus, the longest (4–8 Mb) fragments were not identified for USH at all, while the average length of fragments of this class for CRW and PRW was 4.94 and 4.26 Mb, respectively (Figure 4a,b, Supplementary Table S4a,b).

Analysis of overlapping ROH islands in the three breeds revealed common homozygous regions on chromosomes GGA4 and GGA33 (Table 5). Automated analysis using the Biomart tool on Ensembl found no characterized candidate genes in these regions. However, a manual search on the NCBI resource identified long noncoding RNA (lncRNA) regions on chromosome GGA4. On chromosome GGA33, the genomic area identified as the homozygous region shows the absence of a nucleotide reference sequence, which was likely identified as a common ROH segment for all the breeds studied.

### 3.3. Candidate Genes Affected by Selection and QTLs

We accepted the regions identified by at least two methods or in two pairs of breeds as the areas most strongly subjected to selection pressure in different breeds. Accordingly, the 12 partially overlapping genomic regions on eight chromosomes were established that contained 134 genes, including 27 PCGs (Table 6, Supplementary Tables S1–S3).

Using the Chicken QTLdb database [122,123], we searched for QTLs in the identified genomic regions (Table 7, Supplementary Table S5). A total of 524 QTLs associated with conformation, health, productivity, reproductive, and other phenotypic traits were identified.

As follows from Table 7, the largest number of QTLs was identified in USH (507), whereas PRW and CRW had 12 and 5 QTLs, respectively. Moreover, a significant portion of the QTLs were associated with production traits and reproductive characters (475 and 22, respectively). Notably, QTLs associated with indicators of health (2) and physiological processes (7) were identified in USH selected for cold tolerance [62].

## 4. Discussion

### 4.1. Genetic Diversity among the Breeds Studied

The meat industry in general and the poultry sector specifically essentially rely on the evaluation, characterization, and utilization of genetic diversity inherent in various breeds and lines (e.g., [124,125,126,127,128,129]). In this whole-genome resequencing study, we examined the genetic diversity features in the two divergently selected transboundary meat-type breeds, CRW and PRW, used for producing the Smena 8 broiler cross and compared to the aboriginal cold-tolerant Russian breed of USH. The latter was genetically most distant from the two other breeds, as identified by PCA (Figure 2a,b) and confirmed by the admixture/ancestry analysis (Figure 2c). At K = 2, CRW and PRW demonstrated a common ancestry that was different from that of USH, and most likely corresponds to the meat (Asiatic) type in accordance with the evolutionary model of chicken breed origin and formation [67,130]. At K = 3, the genotyped CRW and PRW chickens split into two obvious clusters (Figure 2c). These can be attributed to the meat (CRW) and dual-purpose (PRW) types according to the traditional classification and phenotypic clustering models, as described in Larkina et al. [59] and Kochish et al. [67].

On the other hand, the PRW and, to a lesser degree, CRW chickens showed signs of admixture from the other breeds in the explored dataset. Overall, USH turned out to be more consolidated genetically and less diverse (Table 1) than PRW and CRW. Also, the allelic diversity in PRW was slightly, but significantly, greater than that in CRW. This is likely due to the history of the creation of this breed and may be a consequence of the use of a larger number of ancestral breeds and lines in developing PRW [131] compared to CRW. Originally, CRW descended from the English local game chickens and Asiatic game (Asil, White Malay, and Indian Game) and meat-type (Cochin) breeds. The initial stock of breeds for developing PRW was somewhat more diverse and included chickens of Asiatic (Java Black, Brahma, Cochin White and Cochin Buff), North American (Dominique), and European (White-faced Black Spanish) origins [67]. The unique genetic makeup and diversity peculiarities of USH we established here were well in line with our previous genome-wide surveys of this old Russian breed [62,63] relative to the genomes of CRW, as well as the Orloff Mille Fleur [59,62,132,133,134,135,136], Russian White [50,51,57,58,137,138,139,140], and other chicken breeds [132,141,142,143].

### 4.2. Inbreeding and ROH Characterization

In a long-term breeding experiment in PRW chickens selected for body weight, Harrison et al. [131] established that, even when inbreeding gradually accumulates and reduces genetic diversity, heterozygosity persists to enable additional responses to selection. Judging from the *_U_F*_IS_ inbreeding coefficients in our study, their significant and much larger values in CRW and PRW perhaps resulted from greater selection pressure in these two transboundary meat-type breeds compared to the local primitive USH fowls that have not been subject to strong artificial selection. On the other hand, the significantly higher number of ROH islands found in USH, as compared to the two other breeds, was likely due to a higher level of inbreeding assessed via *F*_ROH_ in this breed (Table 4), which may also be a consequence of the small size of the existing USH population.

In our previous publication [62], where USH and CRW were also studied, USH was superior to three other breeds, including CRW, in terms of *_U_F*_IS_ inbreeding coefficient. A similar overall pattern was observed for ROH-based inbreeding. In this work, *_U_F*_IS_ turned out to be smaller for USH (about four times less than for CRW and PRW). It was nominally equal to 0.0082, i.e., almost seven times lower than in the previous study (0.055; [62]).

To interpret these apparent inconsistencies in inbreeding estimates, we can assume that this may be due to the chosen genome-wide assessment tool. In the previous article [62], we had an SNP array containing markers that were polymorphic for the breeds on the basis of which it was created. In USH, these same loci could be monomorphic, hence the increased homozygosity. The density of the SNP chip and the size of the examined USH sample can also be important when comparing the two experiments. In the present study, there were about 12.5 M SNPs vs. 50 K on the SNP chip, and the USH sample was smaller (17 vs. 40). We think this may have shifted the *F*_ROH_ inbreeding coefficients in the current survey compared with the data in Romanov et al. [62].

In addition, we would also approach the interpretation of *_U_F*_IS_ and *F*_ROH_ results differently. In the first case, we can talk about selection pressure for a limited number of traits, which, in turn, leads to positive selection in favor of polygenic loci involved in the formation of selected traits, increasing homozygosity at these loci [144,145,146,147]. Since the loci are polygenic, an increase in homozygosity, in most cases, will not be associated with the formation of homozygous haplotypes (only for SNPs located at close distances, that is, due to the hitchhiking effect). However, we introduced a minimum ROH length filter (0.5 Mb) just to exclude short segments resulting from concatenation. In the case of USH, there is no selection and there is virtually no selection pressure. This means that almost all USH individuals produced during the population propagation are left in the next generation, with the exception of the very weak. Moreover, *F*_ROH_ is a more realistic measure of inbreeding. That is, the greater number of longer ROHs in USH (Figure 4b) suggests that this breed has been subject to a more recent inbreeding. This is not surprising, as the USH population has been maintained at 100–200 hens and about 25 roosters for many years, suggesting a higher likelihood of inbreeding. The two commercial breeds surveyed, CRW and PRW, are significantly more numerous and are maintained with over 1000 birds in each line, i.e., over 2000 per breed, hence their lower inbreeding degree. Thus, because *F*_ROH_ is calculated directly from the genome homozygosity of individuals, it provides a more accurate estimate of the inbreeding status within a breed.

### 4.3. Prioritized Candidate Genes within Selective Sweeps

The 12 genomic regions containing the identified selection footprints harbored a total of 27 PCGs that will be described below by chromosome and in terms of their relevance to economically and physiologically important traits in the breeds and lines studied. Notably, this study was consistent with our previous findings presented by Abdelmanova et al. [58] and Romanov et al. [62] for eight selective sweep regions on GGA1, GGA4, GGA5, GGA7, GGA10, and GGA28 in the genomes of CRW, USH, RUW, and OMF chickens (Table 6). On the other hand, we discovered four new genomic regions under selection pressures on GGA2 and GGA14.

#### 4.3.1. GGA1

On this chromosome, we found the *NUAK1* (NUAK family kinase 1) gene known as a potential regulator for chicken plumage pigmentation that overlapped with the respective QTLs [148]. In humans, it is broadly expressed in various tissues, with the highest upregulation in brain [149]. To the best of our knowledge, another nearby gene, *RFX4* (regulatory factor X4), has not been functionally described yet in chickens or other birds. However, its human homolog encodes a testis-specific DNA-binding protein [150] and has a restricted expression toward the brain and, especially, the testis [149], suggesting it as a good reproductive and behavioral candidate gene in chickens.

The *CHST11* (carbohydrate sulfotransferase 11) gene is a reported candidate for plumage color in the chicken that is associated with aggressive behavior, and it is overlapped with the corresponding QTLs [151]. It is also a strong candidate gene for body weight at 35 days in broiler chickens [152], which is also relevant for our study. This gene has a ubiquitous expression in human tissues [149]. The CHST11 enzyme is responsible for catalyzing the chondroitin sulfate that is found on the surface of many cells and extracellular matrix and is the main proteoglycan in cartilage, which might also be important for broiler growth and development. On GGA1, we also identified another important growth and development candidate factor, *IGF1* (insulin like growth factor 1). Previously, it was linked to a signal of selective sweeps, being associated with abdominal fat weight/deposition, body weight, and other traits in chickens [153,154,155,156]. Its human homolog was recognized by a broad expression in various tissues [149].

#### 4.3.2. GGA2

Among the PCGs found on this chromosome, there was *C18orf63,* which encodes an uncharacterized protein in chickens. However, its human homolog (chromosome 18 open reading frame 63) has a restricted, but very high, expression exclusively toward testis tissue [149], suggesting that *C18orf63* may also play a certain role in chicken reproduction. The *CYB5A* (cytochrome b5 type A, or epididymis secretory sperm binding protein type 1 cyt-b5) gene is related to heme binding. In humans, it demonstrates a broad expression in various tissues, especially in liver and kidney [149]. Additionally, it is also described as a rheumatoid arthritis susceptibility gene and is also involved in androgen synthesis [157], thus being supposedly important for functioning of skeletal and reproductive systems. The third PCG revealed on GGA2 was *CCDC102B* (coiled-coil domain containing 102B). It enables protein binding and shows a broad expression in placenta, lung, and other human tissues [149].

#### 4.3.3. GGA4

This chromosome also encapsulates several significant PCGs. One of them, *PCDH7* (protocadherin 7), is a positional candidate gene associated with internal organ traits in chickens and located within a QTL for intestine length and gizzard weight; it is differentially expressed in the epidermis of the feather bud [158,159]. In human tissues, it is relevant to calcium ion binding and cell adhesion and shows a broad expression, especially in the brain [149].

*LCORL* (ligand dependent nuclear receptor corepressor like) is a candidate gene associated with slaughter traits, being positionally associated with internal organ traits in chickens and located within a QTL for intestine length and gizzard weight. It is also a possible candidate responsible for growth and body weight and a reported candidate gene for carcass and eviscerated weight and egg quality traits [57,158,160,161,162,163]. Its human homolog is involved in spermatogenesis, skeletal frame size, and adult height, with a ubiquitous expression in different tissues and the most upregulation in the testis [149]. The *NCAPG* (non-SMC condensin I complex subunit G) gene is involved in mitotic chromosome condensation and may regulate chicken bone growth and development. It is known as a candidate gene for bone size/mass and slaughter traits, with its SNP being also associated with egg albumen quality and other egg traits [160,163,164,165,166]. *NCAPG* has a broad expression in bone marrow, lymph node, testis, and other human tissues [149]. Because of the high importance of the *NCAPG*-*LCORL* locus due to its association with performance and other phenotypic traits, its genetic variation was previously thoroughly explored in chickens of commercial (selected for egg and meat production), local, and imported fancy breeds [59,60]. This investigation suggested prevailed genotypes and specific LD structure at this locus across the studied breeds depending on their utility type and origin.

#### 4.3.4. GGA7

One PCG, *ANKRD44* (ankyrin repeat domain 44), enabling protein binding was found within a selective sweep on this chromosome. This is a candidate gene for dermatological diseases/conditions and is associated with amino acid changes [167]. In humans, its broad expression was reported in various tissues, especially in the lymph node, appendix, and spleen [149].

#### 4.3.5. GGA10

The *FAM189A1* (family with sequence similarity 189 member A1) gene for an uncharacterized protein located in membrane is still understudied in the chicken. However, its human homolog, *ENTREP2* (endosomal transmembrane epsin interactor 2), is expressed in various tissues, with a biased upregulation in the brain [149]. Another PCG, *TJP1* (tight junction protein 1), related to cell adhesion molecule binding was also shown to be associated with decreased fertility in aged laying breeders [168]. It has a ubiquitous expression in human tissues, especially in the testis, placenta, and brain [149].

#### 4.3.6. GGA14

This chromosome harbors the *KDELR2* (KDEL endoplasmic reticulum protein retention receptor 2) gene. The respective protein enables endoplasmic reticulum retention sequence binding. Human *KDELR2* is associated with osteogenesis disorder [169] and has demonstrated a ubiquitous expression in the placenta, stomach, and other tissues [149]. NUBP1 (nucleotide binding protein 1), involved in ATP-dependent iron–sulfur cluster assembly, is known as a host protein that interacts with duck enteritis virus [170]. The *NUBP1* gene has also a ubiquitous expression in adrenal, heart, lymph node, and other human tissues [149].

*TEKT5* (tektin 5) is a nondescribed gene in chickens; its human homolog, however, is involved in cilium assembly and movement, with a restricted expression toward the testis [149]. One more PCG, *EMP2* (epithelial membrane protein 2), is responsible for the corresponding plasma membrane component. It is slightly expressed in various human tissues, with a biased upregulation in the lung, skin, and esophagus [149].

#### 4.3.7. GGA28

A number of PCGs were revealed within one genomic region affected by putative selection on this chromosome and can represent a relevance and significance for describing economically and physiologically important traits in the breeds and lines studied. Of note, five of them, i.e., *CHERP* (calcium homeostasis endoplasmic reticulum protein), *CALR3* (calreticulin 3), *PTPRS* (protein tyrosine phosphatase, receptor type S), *KLF2* (Kruppel like factor 2), and *RAB8A* (RAB8A, member of the RAS oncogene family), have been established as candidates for plasma very-low-density lipoprotein concentration in the chicken [171]. In addition, the *CHERP* gene is known for enabling transmembrane transporter binding activity and RNA binding. It is also typified by ubiquitous expression in human tissues, especially in the testis, spleen, and ovary [149]. *CALR3* is responsible for the respective protein that participates in calcium ion binding and may be associated with obesity in chickens [172]. Its human homolog is marked by a restricted expression exclusively toward the testis [149]. The *PTPRS* gene involved in protein binding and dephosphorylation also has a broad expression in human tissues, especially in fat, the brain, and the prostate [149]. *KLF2* involved in regulation of transcription by RNA polymerase II is additionally related to angiogenesis at tibial lesions in broilers, is considered as a chick connective-tissue-associated transcription factor, and may partly inhibit chicken adipogenesis [173,174,175]. Its human homolog plays roles in many processes during development and disease and is recognized by a broad expression in various tissues, especially in fat and the ovary [149]. The *RAB8A* gene facilitates GTP binding and is distinguished by ubiquitous expression in human tissues, with a higher activity in digestive and immune systems [149].

We also discovered some other PCGs on GGA28. In particular, *C19orf44*, which encodes an uncharacterized chicken protein, is a homolog to the human *C19orf44* (chromosome 19 open reading frame 44) gene, with the latter being defined by ubiquitous expression in different human tissues, especially in the testis and ovary [149]. The *FAM32A* (family with sequence similarity 32 member A) gene product is localized in the nucleolus, being involved in RNA binding activity and, presumably, the apoptotic process. Its human homolog is characterized by ubiquitous expression in digestive and excretory systems and in other tissues [149]. The protein encoded by the *CIB3* (calcium and integrin binding family member 3) gene enables calcium ion binding and has a low expression in human tissues, with a slightly higher expression in the testis [149]. The *TPM4* (tropomyosin 4) gene is related to actin filament binding and muscle contraction, with ubiquitous expression in human tissues and a higher synthesis level in gall and urinary bladders [149]. The *TINCR* (TINCR ubiquitin domain containing) gene is a part of the protein binding pathway and is expressed in several human tissues, with a biased upregulation in the skin, placenta, and esophagus [149].

In summation, the above description of the identified chicken PCGs and the respective functional projection from their human homologs suggest their relevance for artificial selection signatures in the genomes of the transboundary broiler breeds, CRW and PRW, and the primitive native breed of USH. In terms of functionality and association, the 27 PCGs can be attributed to such key economically and physiologically important trait clusters as body weight (*CHST11*, *IGF1*, *LCORL*), growth and development (*IGF1*, *CYB5A*, *PCDH7*, *LCORL*, *NCAPG*, *KDELR2*, *KLF2*), muscles (*TPM4*), fat metabolism and deposition (*IGF1*, *CHERP*, *CALR3*, *PTPRS*, *KLF2*, *RAB8A*), exterior (*NUAK1*, *CHST11*, *PCDH7*, *ANKRD44*, *EMP2*, *TINCR*), behavior (*NUAK1*, *RFX4*, *CHST11*, *PCDH7*, *FAM189A1*, *TJP1*, *TEKT5*, *PTPRS*), immunity (*NCAPG*, *ANKRD44*, *KDELR2*, *NUBP1*, *CHERP*, *KLF2*, *RAB8A*), reproduction (*RFX4*, *C18orf63*, *CYB5A*, *CCDC102B*, *LCORL*, *NCAPG*, *TJP1*, *KDELR2*, *TEKT5*, *CHERP*, *CALR3*, *PTPRS*, *C19orf44*, *CIB3*, *TINCR*), circulatory (*NUBP1*, *TEKT5*), digestive (*KDELR2*, *EMP2*, *RAB8A*, *FAM32A*, *TPM4*, *TINCR*), excretory (*CYB5A*, *NUBP1, FAM32A*, *TPM4*), and respiratory (*CCDC102B*, *EMP2*) systems (Table 6). These results largely overlap with the QTLs found within the determined genomic regions (Table 7, Supplementary Table S5).

Most sweep regions and the conforming 15 PCGs were shared between two or three breeds, suggesting possible similar selective pressure trajectories in their selection history. Partially, this sharing pattern might also be due to common ancestral breeds used for the formation of the three chicken composite breeds studied and occasional gene introgression [176,177], as can be seen from their peculiar admixture-based plots in our investigation (Figure 2c). On the other hand, there were 12 PCGs mostly specific for one breed, PRW, and especially USH, that may reflect certain differences in their distinct genomic architecture. Further in-depth studies will be required to validate shared and breed-specific PCGs that can be linked to the traits under selection pressure.

## 5. Conclusions

In this study, we examined the genomic architecture and diversity of the grandparent lines subject to high selection pressure for meat production and, contrastingly, in an aboriginal Russian chicken breed of USH using whole-genome resequencing data. Probably because of a small population size and peculiar breed history, USH was less heterozygous and diverse and showed a higher genetic distinctiveness relative to two commercial broiler breeds, CRW and PRW. We also dissected 12 regions of selective signatures and the respective candidate genes in these three breeds. To reveal regions under selective pressure, we employed three techniques based on Z*F*_ST_ estimation, hapFLK procedure, and ROH assessment.

The description of the found PCGs in chickens and the corresponding functional projection from human homologs point out that these genes may be relevant for signals of artificial selection seen in the genomes of the transboundary broiler breeds, CRW and PRW, as well as the old local USH breed. Functionally, the 27 PCGs can be associated with important trait clusters that are both physiologically and economically significant, including body weight (*CHST11*, *IGF1*, *LCORL*), growth and development (*CYB5A*, *PCDH7*, *NCAPG*, *KDELR2*, *KLF2*, etc.), muscles (*TPM4*), fat metabolism and deposition (*CHERP*, *CALR3*, *PTPRS*, *RAB8A*, etc.), exterior (*NUAK1*, *ANKRD44*, *EMP2*, *TINCR*, etc.), behavior (*RFX4*, *FAM189A1*, *TJP1*, *TEKT5*, etc.), immunity (*NUBP1*, etc.), reproduction (*C18orf63*, etc.), and digestion, as well as circulatory, excretory, and respiratory systems. The majority of these findings coincide with the QTLs present in the identified chromosomal areas. The information reported here will serve as the basis for detailing the genomic architecture and selection footprints in these breeds and lines and further implementing genomic selection aimed at improving productive and other phenotypic traits in chickens [178,179,180].

## Figures and Tables

**Table 1:** Genetic diversity in the three studied breeds using the basic descriptive statistics ^1^.

Breed ^2^	*n*	*H_O_* (M ± SE)	*_U_H_E_* (M ± SE)	*A_R_* (M ± SE)	*_U_F*_IS_ [CI 95%]
CRW	20	0.2958 ± 0.0001	0.3034 ± 0.0001	1.9101 ± 0.0002 ^a^	0.0363 [0.0358; 0.0368]
PRW	20	0.2958 ± 0.0001	0.3022 ± 0.0001	1.9187 ± 0.0002 ^b^	0.0321 [0.0316; 0.0326]
USH	17	0.2103 ± 0.0002 ^c^	0.2068 ± 0.0001 ^c^	1.6218 ± 0.0004 ^c^	0.0082 [0.0075; 0.0089]

^1^ *n*, number of individuals; *H_O_*, observed heterozygosity; M, mean value; SE, standard error; *_U_H_E_*, unbiased expected heterozygosity; *A_R_*, rarefied allelic richness; *_U_F*_IS_, unbiased inbreeding coefficient (CI 95%, range variation of coefficient at a confidence interval of 95%). ^2^ Breeds: CRW, Cornish White; PRW, Plymouth Rock White; USH, Ushanka. The significance of pairwise values within a column is indicated by different superscripts: ^a^ CRW vs. PRW or USH, *p* < 0.001; ^b^ PRW vs. CRW or USH, *p* < 0.001; ^c^ USH vs. CRW or PRW, *p* < 0.001.

**Table 2:** Mean Z*F*_ST_ values and blocks of SNPs joined by two or more top 0.1% neighbored SNPs at pairwise comparison of the three breeds studied ^1^.

Chromosome	Bin Start ^2^	Bin End ^3^	*N* ^4^	Z*F*_ST_	Breed Pairs	Genes
GGA1	54,420,001	54,470,000	729	0.404483	**CRW**/PRW	*CHST11*
GGA1	54,530,001	54,580,000	1040	0.602406	**CRW**/USH	*CHST11*
GGA1	55,310,001	55,360,000	190	0.884104	CRW/**USH**	*IGF1*
GGA1	55,310,001	55,360,000	291	0.597753	PRW/**USH**	*IGF1*
GGA1	55,320,001	55,370,000	196	0.752325	CRW/**USH**	*IGF1*
GGA1	55,320,001	55,370,000	276	0.568095	PRW/**USH**	*IGF1*
GGA1	75,490,001	75,540,000	148	0.622485	CRW/USH	*TEAD4*
GGA1	75,490,001	75,540,000	157	0.565816	PRW/**USH**	*TEAD4*
GGA1	75,500,001	75,550,000	132	0.663862	CRW/**USH**	*TEAD4*
GGA1	75,500,001	75,550,000	149	0.563412	**PRW**/USH	*TEAD4*
GGA1	75,510,001	75,560,000	67	0.386978	CRW/**PRW**	*TEAD4*
GGA1	75,510,001	75,560,000	125	0.714440	CRW/**USH**	*TEAD4*
GGA1	75,510,001	75,560,000	138	0.579394	PRW/**USH**	*TEAD4*
GGA1	75,520,001	75,570,000	77	0.372578	**CRW**/PRW	*TEAD4*
GGA1	75,520,001	75,570,000	130	0.637783	**CRW**/USH	*TEAD4*
GGA1	188,000,001	188,050,000	477	0.775700	**PRW**/USH	*GRM5*
GGA1	188,010,001	188,060,000	872	0.366252	CRW/**PRW**	*GRM5*
GGA1	188,010,001	188,060,000	548	0.697367	**PRW**/USH	*GRM5*
GGA1	188,020,001	188,070,000	865	0.371221	CRW/**PRW**	*GRM5*
GGA1	188,020,001	188,070,000	596	0.649595	**PRW**/USH	*GRM5*
GGA1	188,030,001	188,080,000	866	0.35713	CRW/**PRW**	*GRM5*
GGA1	188,030,001	188,080,000	649	0.592529	**PRW**/USH	*GRM5*
GGA2	93,720,001	93,770,000	344	0.619266	CRW/**USH**	*CCDC102B*
GGA2	93,720,001	93,770,000	360	0.599763	PRW/**USH**	*CCDC102B*
GGA11	140,001	190,000	362	0.432514	**CRW**/PRW	*SMPD3*
GGA11	140,001	190,000	358	0.603890	**CRW**/USH	*SMPD3*

^1^ Breeds: CRW, Cornish White; PRW, Plymouth Rock White; USH, Ushanka. ^2^ Bin start, start position of sliding window; ^3^ Bin end, end position of sliding window; ^4^ *N*, number of SNP variants in a window. The breed for which a region was determined by comparison with each of the other two breeds is given in bold. Regions identified by pairwise comparison of two breeds are highlighted in color as follows: CRW vs. PRW (red), CRW vs. USH (green), PRW vs. USH (blue).

**Table 3:** HapFLK blocks revealed in the genomes of the studied chicken breeds ^1^.

Chromosome	Breed	Position		Length, Mb	No. of SNPs	Most Significant SNP	Genes
		Start	End				
GGA1	CRW	53,119,864	53,212,505	0.093	82	rs15269046	*SYN3*, ***TIMP3***
	PRW	53,637,245	54,504,503	0.867	569	rs314634881	*NUAK1*, *C12orf75*, *MTERF2, TMEM263, RIC8B*, ***RFX4***, *POLR3B*, *CRY1*, *APPL2*, *WASHC4*, *ALDH1L2*, *SLC41A2*, *CHST11*, *TCP11 × 2*, *CKAP4*, *gga-mir-12210*
GGA6	USH	8,693,825	8,814,126	0.120	108	rs315872719	***KROX20***, *ADO*
GGA16	USH	2,090,051	2,170,380	0.080	190	rs737045576	*IL4I1*, *TRIM7.1*, *SLURP1*, *TRIM39.2*, *TRIM27.2*, *TRIM39.1*, *TRIM27.1*, *TRIM41*, ***RACK1***, *BG1*
	USH	2,230,563	2,248,418	0.018	95	rs740720869	***CENPA***, *CYP21A1*
GGA31	PRW	626,104	665,706	0.040	421	31:6,534,09	–

^1^ Breeds: CRW, Cornish White; PRW, Plymouth Rock White; USH, Ushanka. SNP significance level: *p* < 1 × 10^−5^. Genes in or near which the most significant SNP in the region is located are highlighted in bold. Genes identified at a significance level of *p* < 2.8 × 10^−8^ are underlined.

**Table 4:** The descriptive statistics summary of the homozygosity runs (ROH) ^1^.

Breed ^2^	*n*	ROH Length, Mb			ROH No.			*F*_ROH_		
		M ± SE	Min	Max	M ± SE	Min	Max	M ± SE	Min	Max
CRW	20	102.59 ± 5.71	52.06	147.61	123 ± 6.75	62	164	0.108 ± 0.006	0.06	0.16
PRW	20	95.25 ± 7.54	36.81	155.47	116.45 ± 8.91	50	190	0.101 ± 0.008	0.04	0.16
USH	17	338.75 ± 9.31	262.60	394.09	390.94 ± 9.47	306	435	0.358 ± 0.010	0.28	0.42

^1^ *n*, number of individuals; ROH Length, the overall length of ROHs in a genome; ROH No., the number of ROHs in a genome; *F*_ROH_, inbreeding coefficient calculated based on ROHs; M, mean value; SE, standard error; min, minimal value; and max, maximal value. ^2^ Breeds: CRW, Cornish White; PRW, Plymouth Rock White; USH, Ushanka.

**Table 5:** The ROH islands overlapping in two or more breeds.

Chromosome	Position		Length, Mb	Breed ^1^	Genes
	Start	End			
GGA4	70,462,265	70,739,807	0.278	CRW	*ENSGALG00010011849*, *ENSGALG00010011854*
	70,701,554	70,970,711	0.269	USH	*ENSGALG00010011854*, *ENSGALG00010011667*, *ENSGALG00010011863*, *ENSGALG00010011687*
	70,740,151	71,008,491	0.268	CRW	*ENSGALG00010011667*, *ENSGALG00010011863*, *ENSGALG00010011687*
	70,740,151	70,753,263	0.013	PRW	*ENSGALG00010011667*
GGA33	245,471	1,033,316	0.788	PRW	–
	245,535	1,033,647	0.788	CRW	–
	245,535	1,033,347	0.788	USH	–

^1^ Breeds: CRW, Cornish White; PRW, Plymouth Rock White; USH, Ushanka.

**Table 6:** Genes within the overlapped genomic regions affected by putative selection in the studied chicken breeds and identified by at least two methods.

Chromosome	Sequential Region No.	Position		Length, Mb	Breeds ^1^		Method	Genes
		Start	End		This Study	Previous Studies [58,62]		
GGA1	1	53,637,245	54,504,503	0.867	PRW	CRW, USH	hapFLK	***NUAK1***, *C12orf75*, *MTERF2*, *TMEM263*, *RIC8B*, ***RFX4***, *POLR3B*, *CRY1*, *APPL2*, *WASHC4*, *ALDH1L2*, *SLC41A2*, ***CHST11***, *TCP11X2*, *CKAP4*, *gga-mir-12210*
		53,740,001	53,790,000	0.050	CRW, PRW		Z*F*_ST_	*RFX4*
	2	55,266,291	55,354,497	0.088	PRW	CRW, USH	ROH	***IGF1***
		55,280,001	55,330,000	0.050	CRW, USH		Z*F*_ST_	*IGF1*
GGA2	3	91,027,506	92,075,494	1.048	USH	–	ROH	*FAM69C*, ***C18orf63***, ***CYB5A***, *TIMM21*, *ZNF407*, *CNDP1*, *CNDP2U1*, *FBXO15*
		91,520,001	91,570,000	0.050	CRW, USH		Z*F*_ST_	*C18orf63*, *CYB5A*
	4	92,075,780	93,852,482	1.777	USH	–	ROH	*RTTNDOK6*, *TMX3*, *SOCS6*, ***CCDC102B***, *NETO1*, *CBLN2*, *gga-mir-1803*, *gga-mir-1681, gga-mir-6584*
		93,720,001	93,770,000	0.050	CRW, USH		Z*F*_ST_	*CCDC102B*
GGA4	5	70,754,254	71,145,478	0.391	PRW	RUW, CRW	ROH	***PCDH7***
		70,971,231	71,354,713	0.383	USH		ROH	*PCDH7*
		71,140,001	71,190,000	0.050	PRW, USH		Z*F*_ST_	*PCDH7*
	6	74,938,839	75,922,825	0.984	USH	USH, RUW, CRW	ROH	***LCORL***, ***NCAPG***, *MED28*, *LAP3*, *CLRN2*, *QDPR*, *LDB2*
		75,380,001	75,430,000	0.050	PRW, USH		Z*F*_ST_	*LCORL*, *NCAPG*
GGA5	7	30,830,001	30,880,000	0.050	CRW, PRW	CRW, USH, RUW, OMF	Z*F*_ST_	*MEIS2*
		30,830,467	31,703,025	0.873	CRW		ROH	*CDIN1*, *DPH6*, *ZNF770*, *AQR*, *gga-mir-1718*
GGA7	8	9,270,001	9,320,000	0.050	CRW, USH	CRW	Z*F*_ST_	*DNAH7*
		9,281,277	10,029,387	0.748	USH		ROH	*SF3B1*, *STK17B*, *HECW2*, *GTF3C3*, *C7H2ORF66*, *PGAP1*, ***ANKRD44***, *COQ10B*, *HSPD1*, *HSPE1*, *MOB4*, *RFTN2*, *BOLL*, *PLCL1*
		9,670,001	9,720,000	0.050	CRW, USH		Z*F*_ST_	*ANKRD44*
GGA10	9	5,355,392	6,359,502	1.004	USH	USH, OMF	ROH	*LRRC49*, *THSD4*, *BNIP2*, *GTF2A2*, *GCNT3*, *OTUD7A*, *KLF13*, *TRPM1*, *MTMR10*, *FAN1*, *MPHOSPH10*, *MCEE*, *APBA2*, ***FAM189A1***, ***TJP1***, *TARSL2*, *TM2D3*, *ADAL*, *LARP6*, *gga-mir-204-2*, *gga-mir-1574*
		5,920,001	5,970,000	0.050	CRW, PRW		Z*F*_ST_	*FAM189A1*
GGA14	10	8,062,881	8,813,937	0.751	USH	–	ROH	*C14H16ORF52*, *VWA3A*, *SDR42E2*, *EEF2K*, *POLR3E*, *CDR2*, *METTL9*, *IGSF6*, *OTOA*, ***KDELR2***, *RPS15A*, *ARL6IP1*, *SMG1*, *CLEC19A*, *SYT17*, *COQ7*, *TMC7*, *TMC5*, *GDE1*, *CCP110*, *ITPRIPL2*, *gga-mir-1644*
		8,790,001	8,840,000	0.050	PRW, USH		Z*F*_ST_	*KDELR2*, *DAGLB*, *RAC1*
	11	9,118,484	10,172,206	1.054	USH	–	ROH	*CARHSP1*, *PMM2*, *TMEM186*, *ABAT*, *METTL22*, *TMEM114*, *C16orf72*, *USP7*, ***NUBP1***, ***TEKT5***, ***EMP2***, *GRIN2A*
		9,120,001	9,170,000	0.050	PRW, USH		Z*F*_ST_	*NUBP1*, *TEKT5*
GGA28	12	4,740,302	5,396,354	0.656	USH	RUW, CRW	ROH	***CHERP***, ***C19orf44***, ***CALR3***, ***PTPRS***, *KDM4B*, ***KLF2***, *AP1M1*, ***FAM32A***, ***CIB3***, ***RAB8A***, ***TPM4***, ***TINCR***, *DPP9*, *TNFAIP8L1*, *MED26*, *SLC35E1*, *UHRF1*, *TICAM1*, *FEM1A*, *PLIN3*, *gga-mir-7-3*, *gga-mir-6666*, *MYDGF*
		4,760,001	4,810,000	0.050	CRW, USH		Z*F*_ST_	*CHERP*, *C19orf44*, *CALR3*

^1^ Breeds: CRW, Cornish White; PRW, Plymouth Rock White; USH, Ushanka; RUW, Russian White; OMF, Orloff Mille Fleur. Genes identified by more than one method are highlighted in bold.

**Table 7:** Number of QTLs associated with phenotypic traits identified in the most significant regions presumably subject to selection pressure.

Traits	Breeds ^1^						Total
	CRW	CRW/PRW	CRW/USH	PRW	PRW/USH	USH	
Exterior	**2**	**4**	**1**	**6**	**4**	**6**	**23**
Aggressive behavior		2		3			5
Feather density					4	2	6
Feather pecking	2		1	1		2	6
Feather pigmentation		2		2			4
Receiving feather pecking						2	2
Health						**2**	**2**
Campylobacter intestinal colonization						2	2
Physiology			**11**			**4**	**15**
Blood carbon dioxide level						1	1
CO_2_ partial pressure			3			1	4
VLDL cholesterol level			8			2	10
Production	**1**	**3**	**9**	**2**	**265**	**368**	**648**
Abdominal fat percentage						2	2
Abdominal fat weight		2		2		1	5
Albumen height					6	2	8
Average daily gain					16	69	85
Body slope length						1	1
Body weight	1	1			27	107	136
Body weight gain						1	1
Bursa of Fabricius weight						3	3
Carcass fat content			3			1	4
Carcass weight						12	12
Chest width			4			1	5
Claw percentage						2	2
Claw weight						8	8
Drumstick and thigh muscle percentage					1		1
Drumstick and thigh muscle weight					1		1
Drumstick and thigh percentage					1		1
Drumstick and thigh weight						1	1
Egg number					1	2	3
Egg production rate					3	1	4
Egg weight					128	63	191
Eggshell weight						1	1
Feed conversion ratio			2		12	46	60
Feed intake						3	3
Feet weight						5	5
Femur area					3	1	4
Femur length					3	1	4
Gizzard weight					12	5	17
Head weight						1	1
Heart weight					12	5	17
Liver weight					12	5	17
Muscle dry matter content						1	1
Proventriculus weight					12	5	17
Shank diameter						1	1
Shank length					3	3	6
Spleen weight						1	1
Tibia length					3	2	5
Tibia weight					3	2	5
Wing weight						1	1
Yolk weight					6	2	8
Reproduction					**28**	**13**	**41**
Oviduct length					12	5	17
Oviduct weight					16	8	24
Total	**3**	**7**	**21**	**8**	**297**	**393**	**729**

^1^ Breeds: CRW, Cornish White; PRW, Plymouth Rock White; USH, Ushanka.

## Data Availability

The original contributions presented in the study are included in the article and Supplementary Materials, further inquiries can be directed to the corresponding authors with the permission provided by the chickens’ owners.

## Supplementary Materials

The following supporting information can be downloaded at: https://www.mdpi.com/article/10.3390/genes15040524/s1, Figure S1: Estimation of the number of assumed ancestral populations (K) on the basis of the lowest CV error; Figure S2: The relationship between the LD correlation coefficient and the distance between marker pairs; Figure S3: Manhattan plots for genomic distribution of Z*F*_ST_ values estimated between the CRW, PRW, and USH breeds; Figure S4: Plots of the chromosome areas containing the hapFLK regions; Figure S5: The percentage of SNPs within an ROH island on chromosomes GGA1–GGA15, GGA17–GGA24, GGA26–GGA28, GGA33, and GGA34; Table S1: Mean Z*F*_ST_ values and blocks of SNPs joined by two or more top 0.1% neighbored SNPs at pairwise comparison of the three breeds; Table S2: HapFLK regions identified in genome of the studied chicken populations; Table S3: ROH islands identified in genome of the studied chicken populations; Table S4: Number (a) and overall length of ROHs (b) by ROH length class; Table S5: QTLs detected in identified regions.
