# Supplementary material for: Dissecting Selective Signatures and Candidate Genes in Grandparent Lines Subject to High Selection Pressure for Broiler Production and in a Local Russian Chicken Breed of Ushanka
Source: Genes (Basel). 2024 Apr 22;15(4):524. doi: 10.3390/genes15040524 (PMC11050503; doi:10.3390/genes15040524)
Supplement: Supplementary file 1 [file genes-15-00524-s001.zip › Supplementary Figure S1 (best K).pdf]

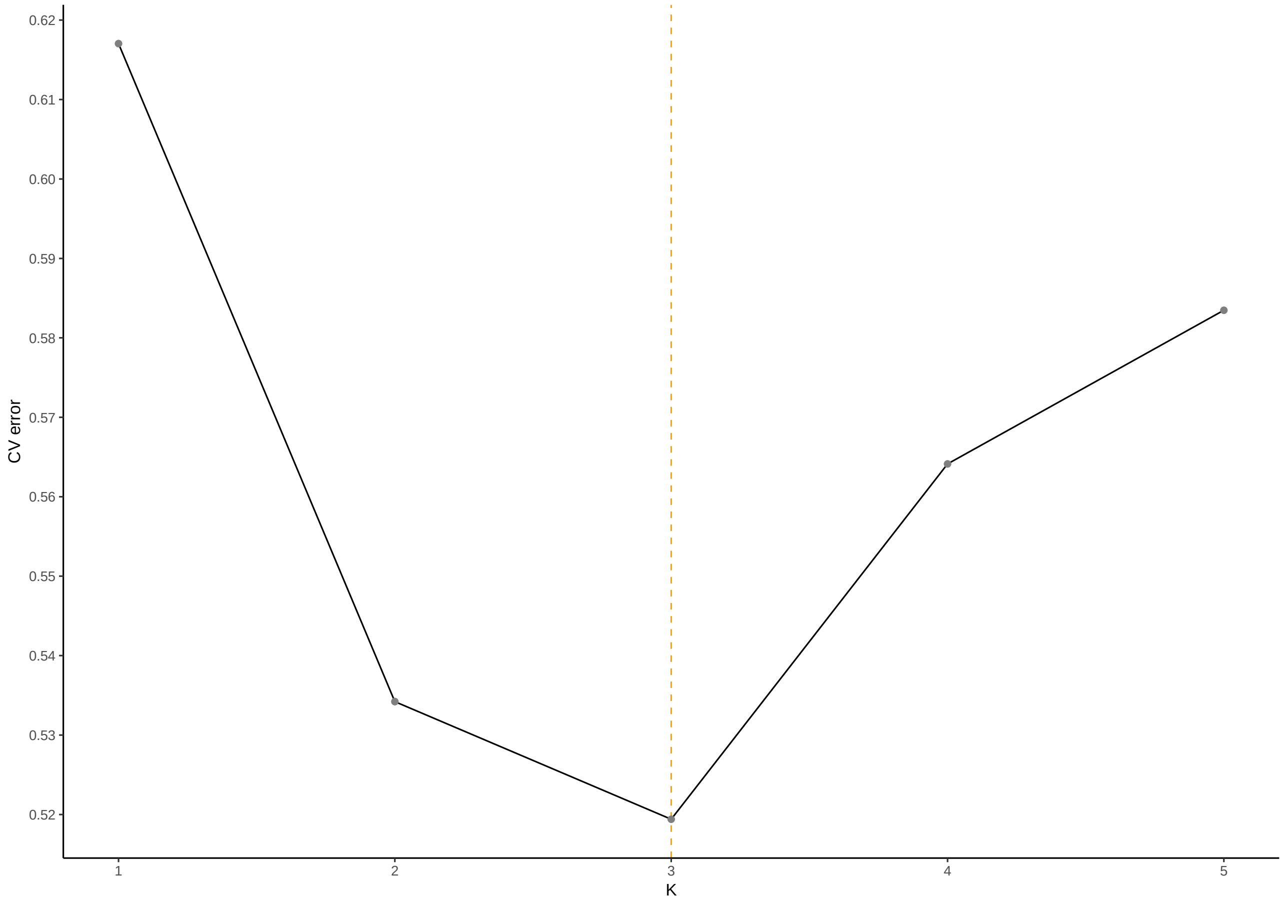

Supplementary Figure S1. Estimation of the number of assumed ancestral populations (K) on the basis of the lowest cross-validation (CV) error. *X*-axis: K, number of ancestral populations; *Y*-axis: values of CV error.
