# Supplementary material for: Dissecting Selective Signatures and Candidate Genes in Grandparent Lines Subject to High Selection Pressure for Broiler Production and in a Local Russian Chicken Breed of Ushanka
Source: Genes (Basel). 2024 Apr 22;15(4):524. doi: 10.3390/genes15040524 (PMC11050503; doi:10.3390/genes15040524)
Supplement: Supplementary file 1 [file genes-15-00524-s001.zip › Supplementary Figure S2 (LD decay 50K).pdf]

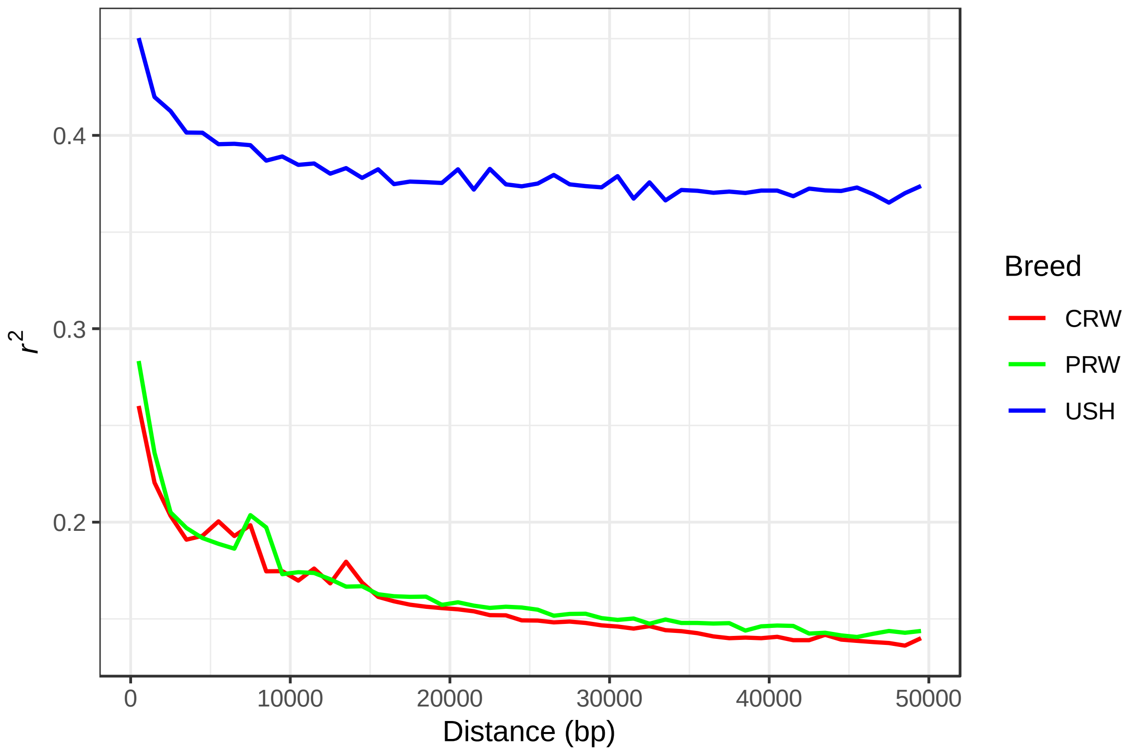

Supplementary Figure S2. The relationship between the linkage disequilibrium (LD) correlation coefficient and the distance between marker pairs. *X*-axis: genetic distance between marker pairs (bp), *Y*-axis: LD values measured as r^2 between pairs of polymorphic marker loci.
