# Supplementary material for: Dissecting Selective Signatures and Candidate Genes in Grandparent Lines Subject to High Selection Pressure for Broiler Production and in a Local Russian Chicken Breed of Ushanka
Source: Genes (Basel). 2024 Apr 22;15(4):524. doi: 10.3390/genes15040524 (PMC11050503; doi:10.3390/genes15040524)
Supplement: Supplementary file 1 [file genes-15-00524-s001.zip › Supplementary Figure S3 (FST manhattan).pdf]

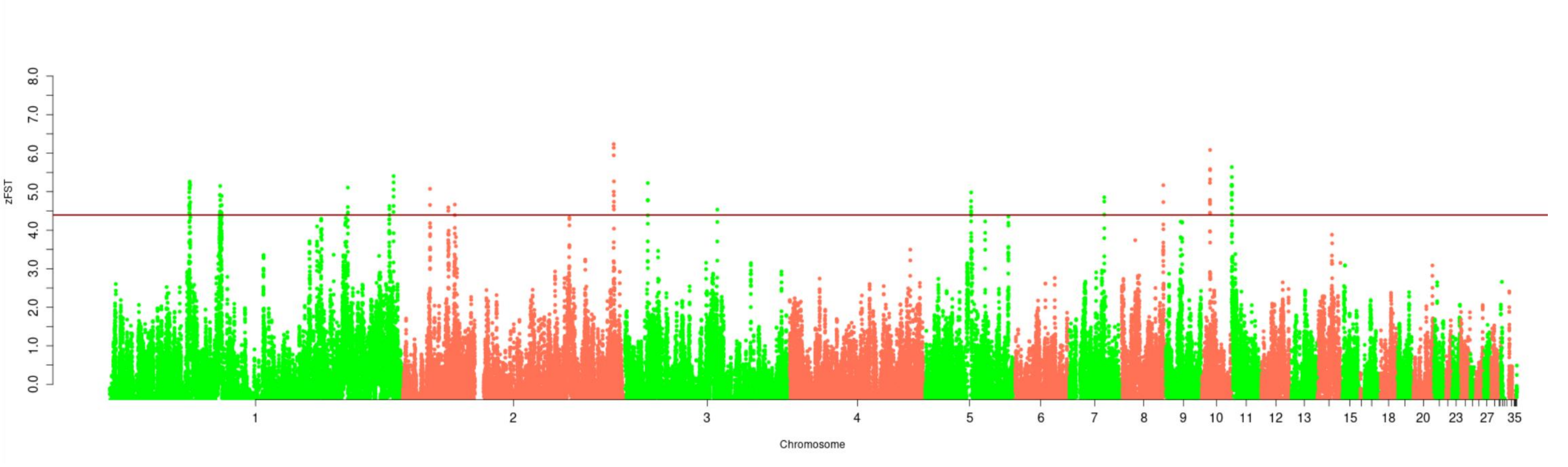

zFST
0.0
1.0
2.0
3.0
4.0
5.0
6.0
7.0
8.0
1
2
3
4
5
6
7
8
9
10
11
12
13
15
18
20
23
27
35
Chromosome

(**a**)

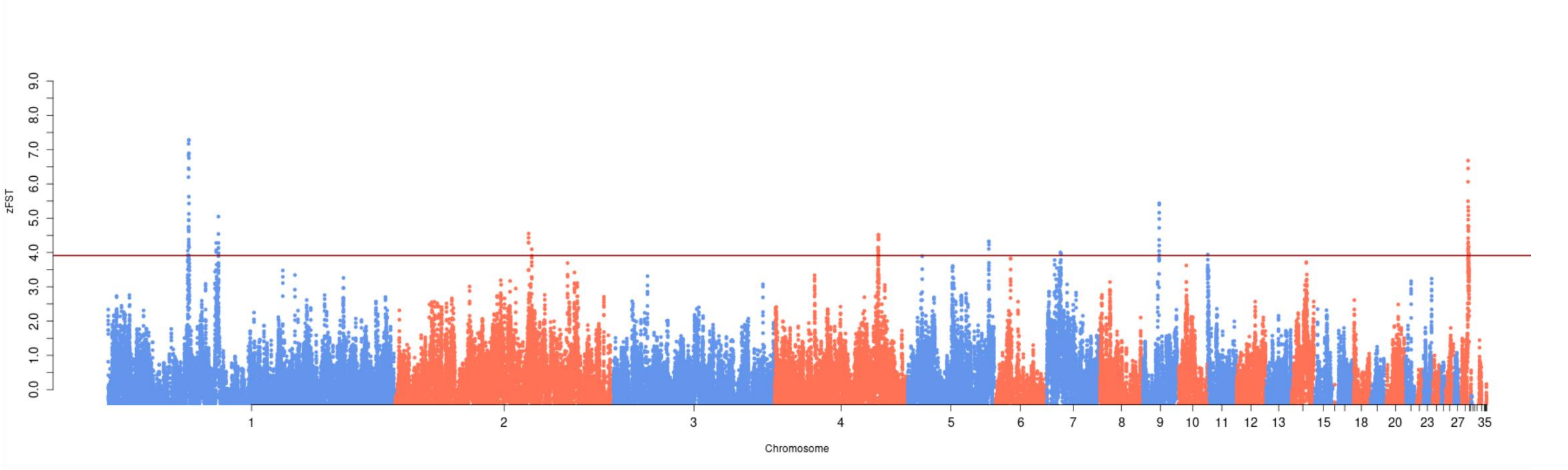

zFST
0.0
1.0
2.0
3.0
4.0
5.0
6.0
7.0
8.0
9.0
1
2
3
4
5
6
7
8
9
10
11
12
13
15
18
20
23
27
35
Chromosome

(**b**)

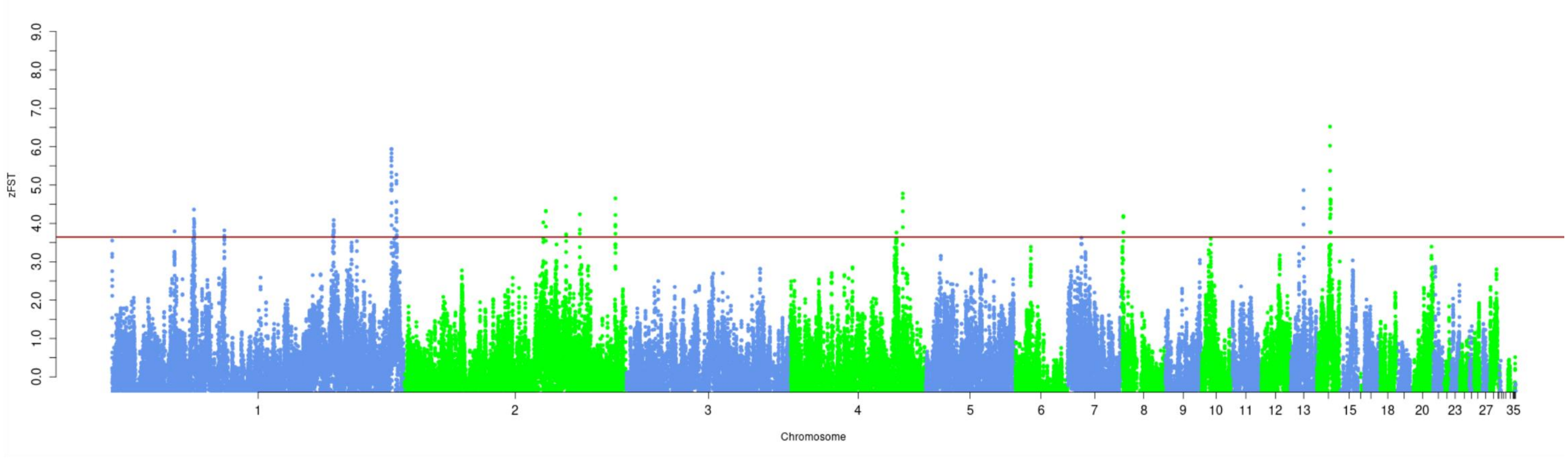

(**c**)

Supplementary Figure S3. Manhattan plots for genomic distribution of zF_{ST} values estimated between the Cornish White (CRW), Plymouth Rock White (PRW) and Ushanka (USH) breeds. Values for the *X*-axis are chicken autosomes (breadth of autosomes corresponds to their length) and those for the *Y*-axis are zF_{ST} values. SNPs were plotted relative to their positions within each autosome. The threshold, which was estimated as the top 0.1% for zF_{ST} values, is indicated by a horizontal line. Breeds: (**a**) CW vs. PRW; (**b**) CW vs. USH; (**c**) PRW vs. USH.
