# Supplementary material for: Dissecting Selective Signatures and Candidate Genes in Grandparent Lines Subject to High Selection Pressure for Broiler Production and in a Local Russian Chicken Breed of Ushanka
Source: Genes (Basel). 2024 Apr 22;15(4):524. doi: 10.3390/genes15040524 (PMC11050503; doi:10.3390/genes15040524)
Supplement: Supplementary file 1 [file genes-15-00524-s001.zip › Supplementary Figure S5 (SNPs in Runs).pdf]

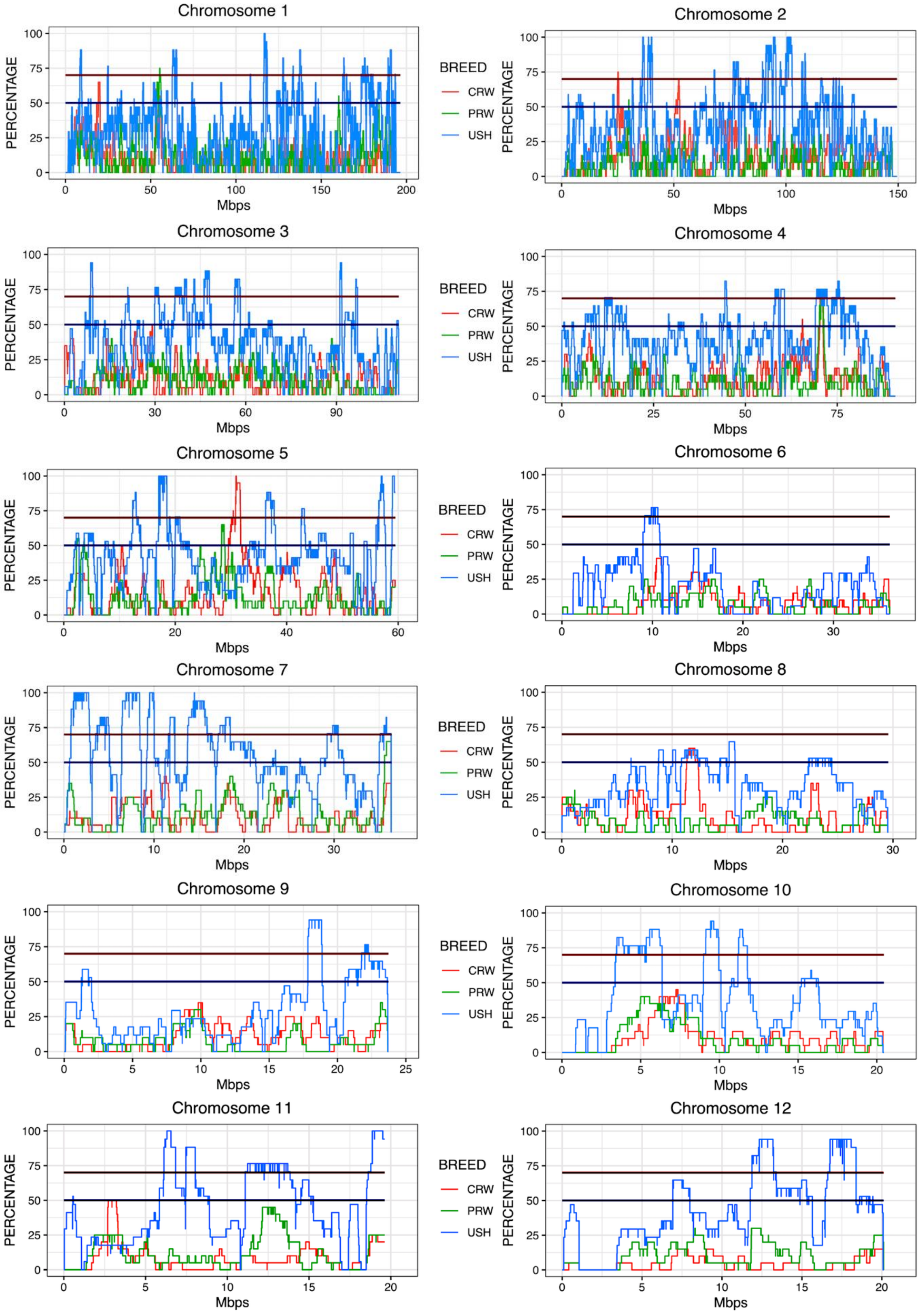

Chromosome 1
Chromosome 2
Chromosome 3
Chromosome 4
Chromosome 5
Chromosome 6
Chromosome 7
Chromosome 8
Chromosome 9
Chromosome 10
Chromosome 11
Chromosome 12
PERCENTAGE
Mbps
BREED
CRW
PRW
USH

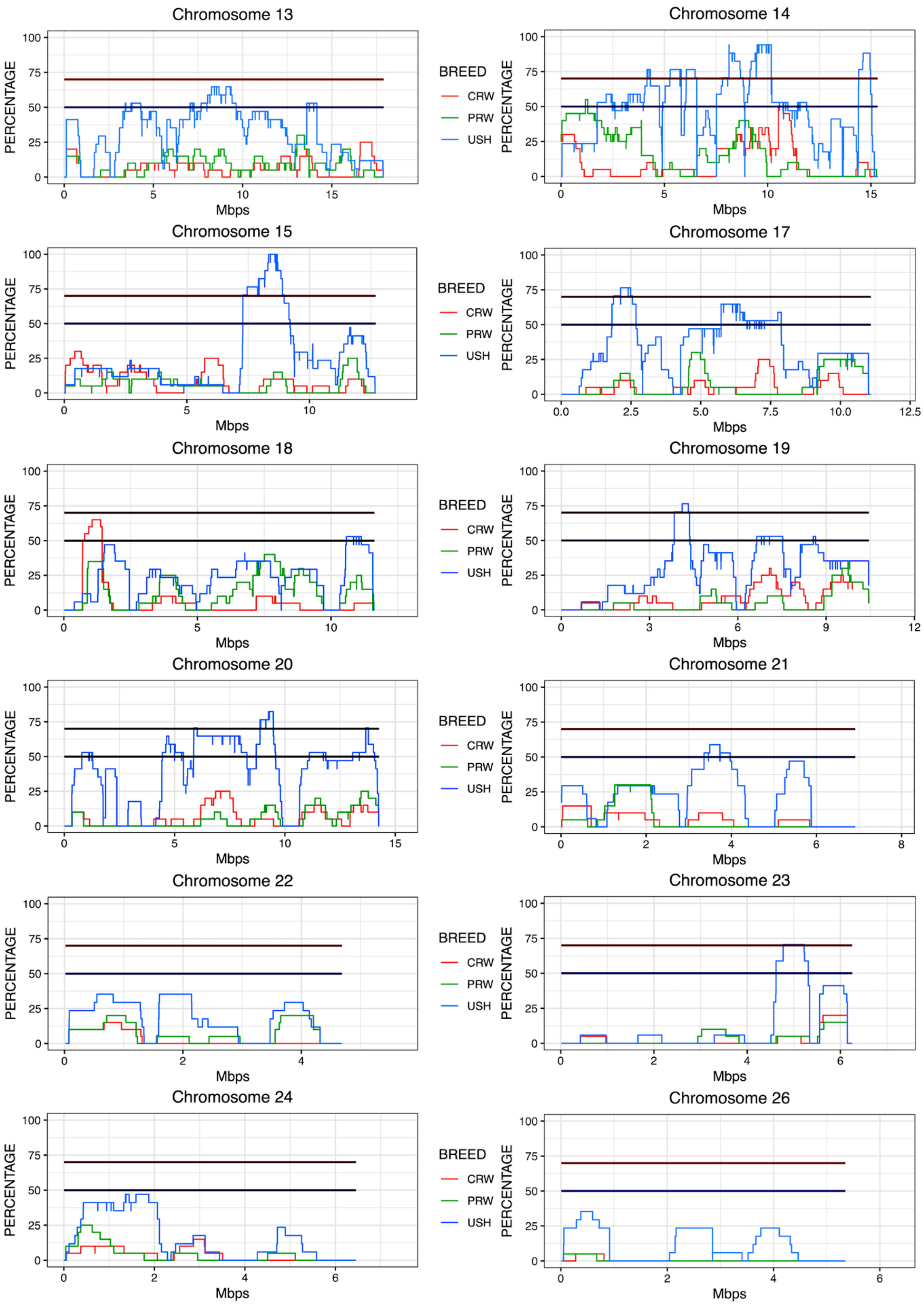

Chromosome 13
Chromosome 14
Chromosome 15
Chromosome 17
Chromosome 18
Chromosome 19
Chromosome 20
Chromosome 21
Chromosome 22
Chromosome 23
Chromosome 24
Chromosome 26
PERCENTAGE
Mbps
BREED
CRW
PRW
USH

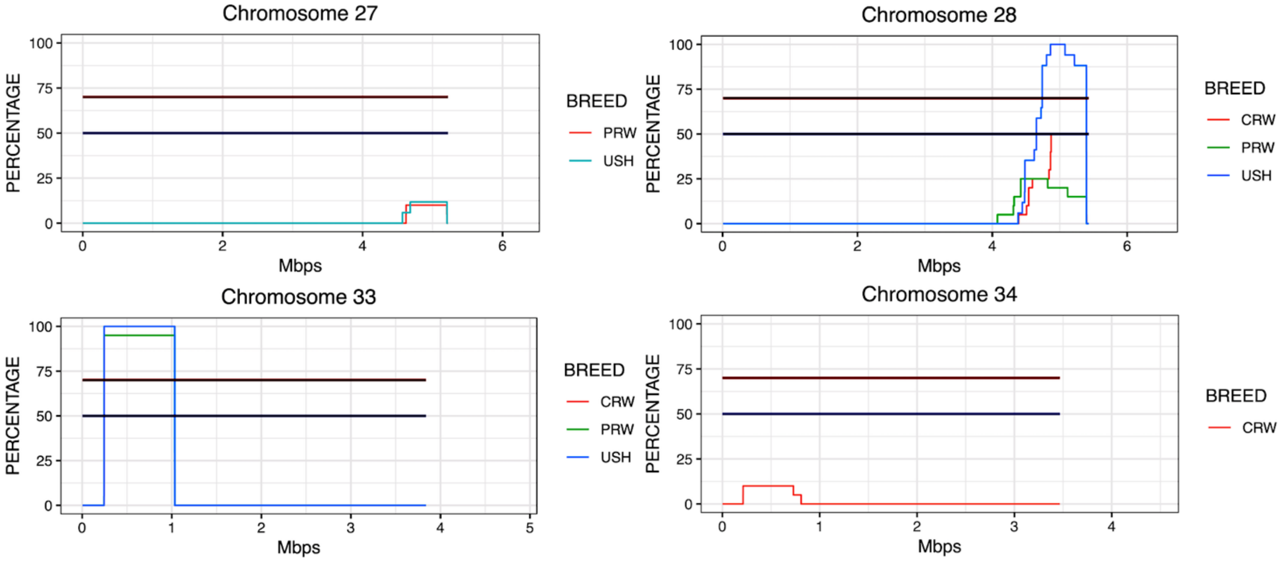

Supplementary Figure S5. The percentage of SNPs within a ROH island on chromosomes GGA1–GGA15, GGA17–GGA24, GGA26–GGA28, GGA33 and GGA34. *X*-axis: genetic distance (Mbps); *Y*-axis: percentage of SNPs detected within a ROH island. Blue line shows 50% threshold, and red line conforms to 70% threshold.
