# Supplementary figures and images for: Dissecting Selective Signatures and Candidate Genes in Grandparent Lines Subject to High Selection Pressure for Broiler Production and in a Local Russian Chicken Breed of Ushanka
Source: Genes (Basel). 2024 Apr 22;15(4):524. doi: 10.3390/genes15040524 (PMC11050503; doi:10.3390/genes15040524)

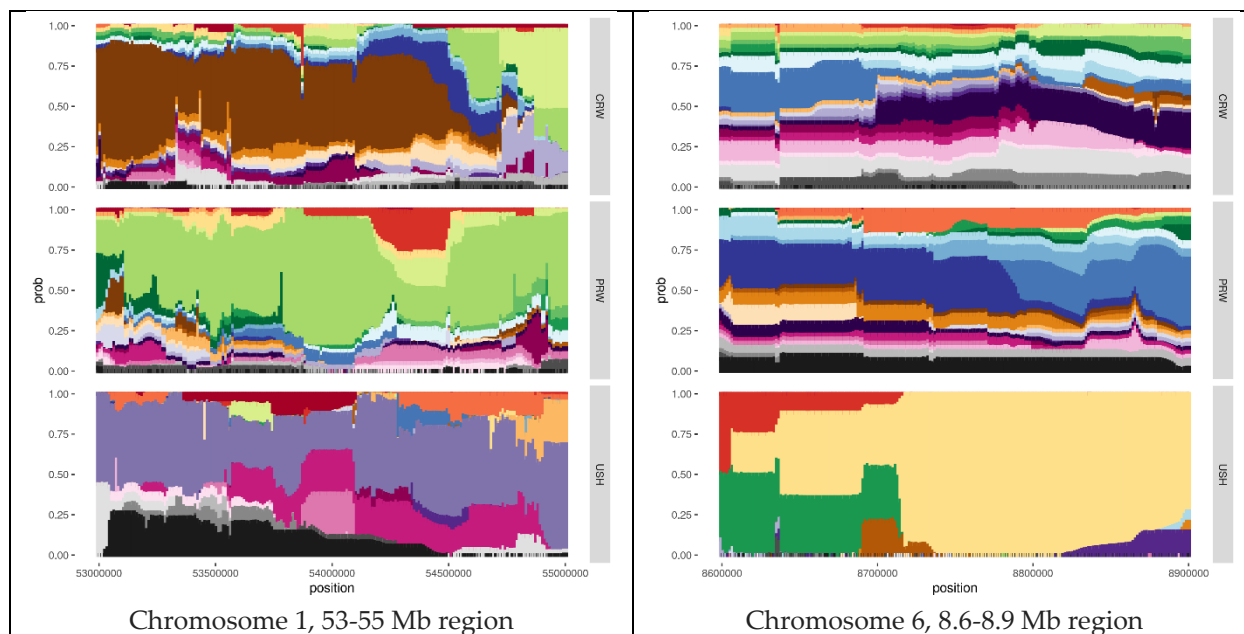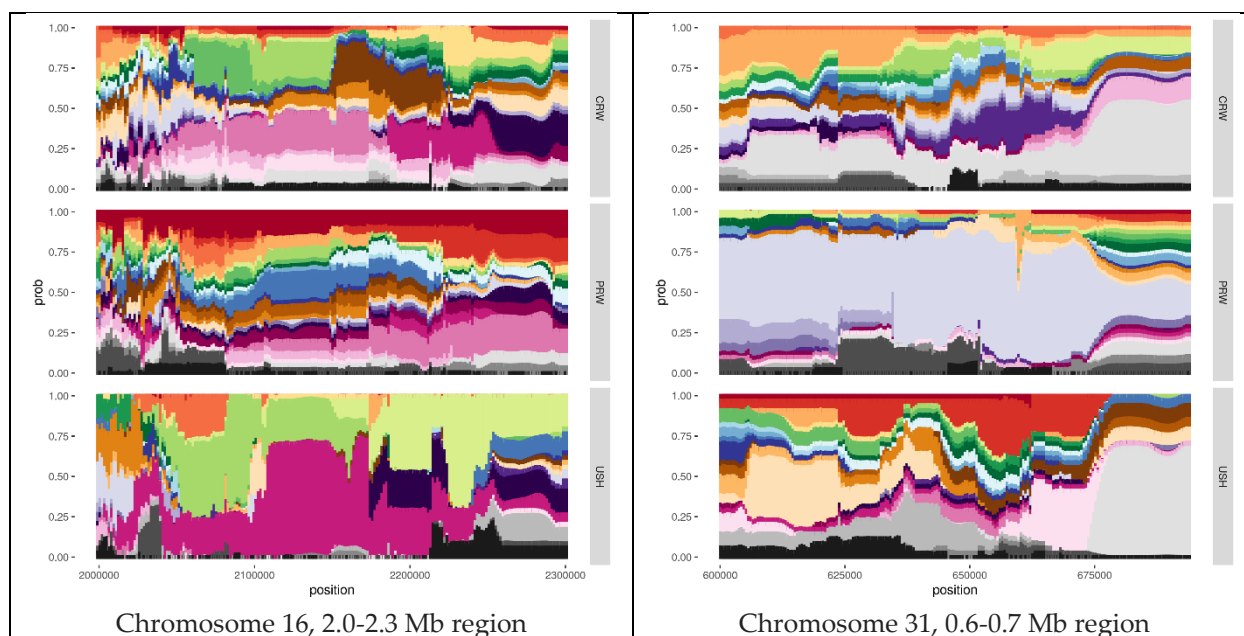

**Supplementary Figure S4.** Plots of the chromosome areas containing the hapFLK regions.

Supplement: Supplementary file 1 [file genes-15-00524-s001.zip › Supplementary Figure S4 (hapFLK).pdf]
